# Single Nucleotide Polymorphisms Associated with MicroRNA Regulation

**DOI:** 10.3390/biom3020287

**Published:** 2013-04-09

**Authors:** Yu Jin, Caroline G. L. Lee

**Affiliations:** 1 NUS Graduate School for Integrative Sciences and Engineering, National University of Singapore, Singapore 117456, Singapore; E-Mail: g0901870@nus.edu.sg; 2 Department of Biochemistry, National University of Singapore, Singapore 119077, Singapore; 3 Division of Medical Sciences, National Cancer Centre, Singapore 169610, Singapore; 4 Duke-NUS Graduate Medical School, Singapore 169547, Singapore

**Keywords:** microRNA, single nucleotide polymorphism, bioinformatics, natural selection, expression phenotype, database

## Abstract

Since the discovery of microRNA (miRNA), the polymorphisms that affect miRNA regulation had been extensively investigated by many independent studies. Recently, researchers utilized bioinformatics and statistical approaches for genome-wide analysis on the human polymorphisms that reside in the miRNA genes, targets, and/or genes involved in miRNA processing. In this review, we will give an overview about the important findings of these studies from three perspectives: architecture of the polymorphisms within miRNAs or their targets, potential functional consequences of the polymorphisms on miRNA processing or targeting, and the associations of the polymorphisms with miRNA or target gene expression. The results of the previous studies demonstrated the signatures of natural selections on the miRNA genes and their targets, and proposed a collection of potentially functional, expression-associated, and/or positively selected polymorphisms that are promising for further investigations. In the meantime, a few useful resources about the polymorphic miRNA regulation have been developed and the different features of these databases were discussed in this review. Though recent research had benefited from these comprehensive studies and resources, there were still gaps in our knowledge about the polymorphisms involved in miRNA regulation, and future investigations were expected to address these questions.

## 1. Introduction

MicroRNAs (miRNAs) are endogenous small non-coding RNAs that are transcribed from genomic sequences, cleaved into miRNA precursors (pre-miRNAs), and further processed into approximately 22-nucleotide mature miRNAs [1]. In animals, miRNAs were found to hybridize to 3’ untranslated regions (3’UTRs) and mediate mRNA cleavage or translational inhibition [2]. To date, there are more than 1000 miRNA transcripts identified in the human genome, according to miRBase database (release 19) [3]. As an important class of regulators, they were reported to be involved in a wide range of biological processes [4].

Since the discovery of miRNAs, a great number of studies had characterized genetic polymorphisms that affect miRNA regulation via different molecular mechanisms and associated with various phenotypic differences. First, polymorphisms within precursor miRNAs (pre-miRNAs) were found to affect miRNA expression possibly via altering pre-miRNA processing [5]. Second, polymorphisms in miRNA target sites had been extensively studied and implicated in a variety of human diseases [6]. Moreover, it had been identified that sequence variations in miRNA machinery genes e.g., DICER had been involved in diseases possibly via altering miRNA-mediated regulation [7]. Independent studies supported the functional importance of polymorphisms associated with miRNA regulation. Nonetheless, there is still a need for systemic and comprehensive investigation of these polymorphisms. 

With the availability of the polymorphism data, researchers began to investigate polymorphic miRNA regulation in a global manner. They conducted both bioinformatics prediction and statistical approaches to elucidate the effects of genetic variations on miRNA regulation and expression phenotypes, and provide possible explanations for the disease associations. Moreover, recent studies also revealed the signatures of natural selections at miRNA loci and targets [8,9,10,11,12]. In this article, we would like to give an overview of the genome-wide investigations on the miRNA-related polymorphisms, identify the gaps in our current knowledge, and propose future studies in this area.

## 2. Architecture of Genetic Polymorphisms Associated with MiRNA Regulation

In the genome-wide investigations of miRNA-related polymorphisms, researchers utilized single nucleotide polymorphism (SNP) data from dbSNP database to elucidate the distribution of genetic variants at miRNA loci and their targets. Saunders *et al.* found that polymorphisms occur less frequently within pre-miRNAs, compared to the flanking sequences. In addition, very few polymorphisms were identified in the seed regions, which were defined as the second to the seventh nucleotides in the mature miRNAs and are critical for target recognition [10]. The results indicated that miRNA genes are selectively constrained. Using more updated information with the increased number of novel SNPs and miRNA genes annotated recently, another study illustrated that miRNA loci, in particular, the seed regions of the mature sequences, are subjected to negative selection, as demonstrated by low SNP density and allele frequency. In contrast to the previous observation that approximately 90% pre-miRNAs were non-polymorphic, the later study identified that above 40% pre-miRNAs contain at least one polymorphism and 48 SNPs were found in the functionally important seed regions [13]. Moreover, by studying the disease-related miRNAs, Lu *et al.* found that SNPs occur less frequently, hence selectively constrained in the miRNAs associated with diseases, compared to the other miRNAs [14]. 

In addition to the polymorphisms at miRNAs, a few studies characterized the distribution of SNPs at miRNA targets. Different from miRNA genes which are well annotated, not all the miRNA targets are experimentally validated and identification of target sites rely on experimental evidence and bioinformatics prediction. Different approaches had been utilized to identify the putative miRNA target sites. Chen *et al.* [9] and Saunders * et al.* [10] searched for conserved 3’UTR sequences with canonical miRNA sites, which were characterized as 7- or 8-mers that match the seed regions of the mature miRNAs. Yu *et al.* [11] utilized the putative sites by PicTar [15] while Hu *et al.* [8] investigated the polymorphisms in the targets predicted by TargetScan [16]. Though different bioinformatics prediction and SNP data were used in these studies, they reached the same conclusion that signatures of purifying selection were identified in the miRNA target sites, compared to the flanking sequences in the 3’UTRs. It suggests that polymorphisms in the miRNA target sites tend to be deleterious and may contribute to disease phenotypes. 

Despite the observation of negative selection at miRNA target sites, Chen *et al.* and Saunders *et al.* identified several SNPs that alter verified or predicted binding sites showing high allele frequency and evidence of recent positive selection, which is reflected by long-range haplotypes and population differentiation [9,10]. Moreover, Richardson *et al.* identified that SNPs potentially altering miRNA binding sites generally exhibit higher levels of population differentiation, compared to the remaining 3’UTR SNPs [12]. All the results above indicated that SNPs at miRNA target sites are more likely to be under positive selection pressure and contribute to differences among populations.

## 3. Potential Functions of Polymorphisms Associated with MiRNA Regulation

Characterizing the functions of the genetic variants associated with miRNAs is a critical part in the studies related to polymorphic miRNA regulation. While experimental approaches were low-throughput and not suitable for genome-wide investigations, researchers utilized bioinformatics tools for predicting potential functions of miRNA-related SNPs. Such studies explained possible molecular mechanisms for genetic variants leading to phenotypic changes, and shed light on future investigation and validation.

### 3.1. Potential Functions of SNPs within miRNAs

An early study by Iwai *et al.* investigated 10 pre-miRNA SNPs that were identified by sequencing 173 pre-miRNAs in 96 Japanese individuals. Based on the locations and sequence changes caused by the SNPs, the authors predicted that three SNPs could affect miRNA biogenesis or target selection [17]. A more comprehensive study conducted by Duan *et al.* included all the SNPs in miRNA loci and predicted their effects on minimum free energy (MFE) of pre-miRNA hairpin structures. It was found that the highest MFE changes are caused by SNPs within mature sequences, followed by SNPs in the stem regions and loop domains [18]. Later, Gong *et al.* collected a list of published pre-miRNA SNPs, including those identified by Duan *et al.* previously, and evaluated their effects on the stability of pre-miRNAs based on the energy change of the hairpin structures. The study showed that 44% of the candidate SNPs cause significant changes in the stem-loop structures and are likely to affect production of mature miRNAs [13]. Similarly, the impact of SNPs on pre-miRNA structures were predicted by Hiard *et al.* and included in the Patrocles database [19].

It had been found that polymorphisms are rare in mature sequences, especially seed regions [9,10]. Jazdzewski *et al.* identified a SNP within miR-146a* altered the expression of mature miRNAs, and could potentially affect target recognition [5,20]. A systemic study conducted by Zorc *et al.* identified the seed polymorphisms in vertebrates, and assessed whether a SNP changes the seed sequence of a mature miRNA to that corresponding to a different miRNA [21]. A few candidate polymorphisms were illustrated in the study, while the downstream effects on the target genes were not investigated. Gong *et al.* identified 48 SNPs in the seed regions and utilized miRanda [22] and TargetScan [16,23] algorithms to predict the changes in miRNA targets. This study demonstrated that SNPs in seed regions lead to creation and disruption of putative binding sites, and the total numbers of putative targets could be drastically altered by these polymorphisms. As a consequence, the transcript expression patterns could be significantly affected by SNPs within miRNAs, and the authors also successfully validated a few candidates using Dual-luciferase assay [13]. Similarly, Ziebarth *et al.* identified the SNPs in the seed regions of the human miRNAs and evaluated their effects on disruption or creation of target sites, which were incorporated in the PolymiRTS 2.0 database [24].

### 3.2. Potential Functions of SNPs within miRNA Targets

SNPs in the miRNA target sites had been comprehensively investigated by a number of studies, which utilized different approaches to assess their potential functions. Though some previous studies revealed that miRNAs could bind to 5’ untranslated regions (5’UTRs) or coding sequences of mRNAs [25,26], thus the genetic variants in these regions potentially affect miRNA regulation, most of the studies still focused on the polymorphisms in 3’UTRs. 

Functional SNPs in the miRNA targets were categorized into two categories according to whether they create or disrupt miRNA binding sites. To predict SNPs disrupting miRNA target sites, several studies identified the canonical 7-mers or 8-mers in 3’UTRs that match the seed regions of miRNAs, and the SNPs in these regions are likely to abolish miRNA bindings [9,10,27,28]. Duan *et al.* utilized TargetScanS [29] and PITA [30] to identify the target sites, and the SNPs in the see-pairing regions predicted by both algorithms were determined to be potentially functional [18]. On the other hand, SNPs that create novel seed sites showed less selective constraints, and some studies required the co-expression of miRNA and their putative targets in the same tissues as a criterion to determine the candidate SNPs to be functionally important [9,10]. 

In addition to identifying SNPs that create or disrupt canonical miRNA binding sites, a few studies relied on the well-developed algorithms for target prediction to assess the potential functions of 3’UTR SNPs. For example, the identification of functional SNPs by Richardson *et al.* was based on the predicted miRNA target seed sites by mirSVR model [31], and the SNPs that reside in these sites potentially affect miRNA bindings. The authors also used the miRanda algorithm [22] to predict whether allele change of a 3’UTR SNP leads to creation of novel binding sites according to the change in the alignment and energy scores [12]. Similarly, Liu *et al.* and Landi *et al.* used the same algorithm for target prediction in the sequences containing SNPs, and identified their potential effects on miRNA targeting according to the differences in the alignment scores and variations in binding free energy, respectively [32,33].

Though the majority of the studies assessed 3’UTR SNPs based on whether they change the sequences of putative target sites, Hariharan *et al.* investigated their effects on the secondary structures of the miRNA binding sites. In this study, the sequences surrounding the 3’UTR SNPs of interests were extracted, and the secondary structures of the sequences with alternative alleles were predicted using RNAfold. The degree of change in the secondary structure indicates the influence on accessibility for miRNA targeting, and therefore is used for prediction of creation or disruption of miRNA binding sites [34]. 

Thomas *et al.* utilized a more complex tool for identifying the candidate 3’UTR SNPs [35]. Their prediction was based on a previously developed two-step support vector machine (SVM) model, which could incorporate a number of local features, for example, base-paring in the seed regions and global features, for example 3’UTR length [36]. The authors assessed whether haplotypes in 3’UTRs are likely to be down-regulated by a particular miRNA. They further identified the significant differences between the haplotypes and shortlisted the candidate SNPs that contribute to such differences. This method was reported to outperform the approaches based on changes on free energy or context score by TargetScan, as it fits better with allelic imbalance sequencing data [35]. 

Last but not least, some studies identified the SNPs in the validated miRNA target sites as potentially functional. Validated target sites from publicly available databases e.g., TarBase [37], miRecords [38] and miRTarBase [39] had been utilized to identify the SNPs that potentially affect miRNA targeting [10,12,18,24]. Furthermore, with the availability of PAR-CLIP (Photoactivatable-Ribonucleoside-Enhanced Crosslinking and Immunoprecipitation) sequencing data [40], SNPs in these experimentally identified miRNA binding sites were also determined to be functionally important [24].

## 4. Polymorphisms Associated with MiRNA or Target Expression

With the availability of genome-wide SNP genotype and transcript expression data, recent studies further investigated the associations between genotypes and expression phenotypes, in addition to the potential functions of genetic variants. With regard to the polymorphisms associated with miRNA regulation, researchers focused on two groups: genetic variants that affect miRNA expression and those that alter transcript abundance via changing miRNA target sites. These studies utilized the genotype and miRNA or mRNA expression data of the same individuals to analyze the associations, and the lymphoblastoid cell lines (LCLs) from healthy individuals of HAPMAP project [42] had been most commonly used for such genetic investigations.

**Table 1:** Summary of Studies on single nucleotide polymorphisms (SNPs) within microRNA (miRNAs).

Study	SNP resource	MiRNA resource	Number of SNPs in pre-miRNAs	Tools for functional predictions of SNPs on	
				miRNA biogenesis and stability	target selection
Iwai *et al.* [17]	Sequencing data	Rfam database	10	analysis based on the locations and the sequence changes by the SNPs	miRanda
Saunders *et al.* [10]	dbSNP126	miRBase release 9	65	-	-
Duan *et al.* [18]	dbSNP129	miRBase release 13	188	RNAfold	-
Hiard *et al.* [19]	dbSNP	miRBase	184	RNAfold	-
Bhartiya *et al.* [41]	dbSNP130	miRBase release 13	106	PHDcleav, RISCbinder	-
Gong *et al.* [13]	dbSNP132	miRBase release 16	757	energy changes curated from literatures	TargetScan, miRanda
Liu *et al.* [32]	dbSNP135	miRBase release 18	1940	-	-

**Table 2:** Summary of Studies on SNPs in miRNA Targets.

Study	3'UTR SNP resource	Tools for identifying SNPs in	
		putative targets	verified targets
Chen *et al.* [9]	dbSNP125	PicTar-like algorithm	
Saunders *et al.* [10]	dbSNP126	TargetScanS	Tarbase
Bao *et al.* [27]	dbSNP126	TargetScanS	-
Yu *et al.* [11]	dbSNP126	PicTar	-
Landi *et al.* [33]	dbSNP	PicTar, DIANA-MicroT, miRBase, miRanda, TargetScan, MicroInspector	-
Hariharan *et al.* [34]	dbSNP123	RNAFold	-
Duan *et al.* [18]	dbSNP129	TargetScanS and PITA	Tarbase
Hiard *et al.* [19]	dbSNP	predicting SNPs that alter canonical sites matching miRNA seed regions, or conserved octamers in 3'UTRs	-
Thomas *et al.* [35]	HapMap Release 22	SVM prediction model	-
Hu *et al.* [8]	dbSNP130	TargetScan	-
Richardson *et al.* [12]	dbSNP132	miRanda	miRecords
Gong *et al.* [13]	dbSNP 132	TargetScan and miRanda	-
Zhang *et al.* [28]	HapMap release 27	predicting SNPs that disrupt or create canonical site motif complementary to seed regions	-
Ziebarth *et al.* [24]	dbSNP132	TargetScanS	Tarbase, MiRecords, miRTarBase, CLIP-sequencing, allelic imbalance sequencing
Liu *et al.* [32]	dbSNP135	miRanda	-

### 4.1. SNPs Associated with miRNA Expression

MiRNA expression in healthy individuals had been characterized by a few studies. Wang *et al.* measured both miRNA and mRNA expression in LCLs from 90 Caucasian males, and identified significant correlations between miRNA and mRNAs [43]. The expression data had not been integrated with genotype data to identify the genetic associations, but provided useful information about the expressed miRNAs in the same cell lines from different individuals [44]. 

A later study by Huang *et al.* measured miRNA expression in LCLs from both CEU (Utah residents with ancestry from northern and western Europe) and YRI (Yoruba in Ibadan, Nigeria) populations of the international HAPMAP project. This study focused on the differentially expressed miRNAs between populations and the genetic variants that could contribute to such differences. The SNPs with high levels of population differentiations were selected and showed significant associations with 36% differentially expressed miRNAs between the two populations. Most of the associations are trans-associations and no functional characterization of the SNPs was conducted [45]. The same group carried out a more comprehensive analysis by including all the expressed miRNAs and common SNPs in the same group of HAPMAP samples [46]. At a genome-wide significance level after multiple test correction, 31 significant associations between SNPs and mature miRNA expression were identified, and consistent with the previous finding, they are mainly trans-associations, and none of the candidate SNPs resides within the mature miRNA sequences. In addition to the expression associations, the study also found that the miRNA-associated SNPs are enriched with SNPs that are associated with mRNA expression and could be related to complex traits and disease risks [46].

### 4.2. SNPs Associated with miRNA Target Expression

In contrast to the limited availability of the miRNA expression data, a greater number of studies had profiled mRNA expression in different tissues of healthy individuals, and identified the expression-associated polymorphisms [47,48,49,50,51]. Integrated analysis of the expression associations and potential functions of the 3’UTR polymorphisms had been conducted by a few recent studies, which identified a number of candidate SNPs that affect gene expression via altering miRNA regulation, and provided evidence for the associations of SNPs with phenotypic differences. 

To obtain a global view about the target site polymorphisms affecting gene expression, the first question is whether the SNPs in miRNA targets could contribute significantly to the expression variations, compared to SNPs in other regions. This question was addressed by Lu *et al.* who analyzed the genotype and mRNA expression in four populations of the HAPMAP project, and found that compared to introns, 3’UTRs contain an excess of SNPs associated with expression phenotypes. Moreover, SNPs that reside in the regions that match to seed sequences of miRNA also have a higher chance of being associated with expression, as compared to intronic SNPs. The results implied that functional SNPs in the 3’UTR are likely to alter gene expression via affecting miRNA targeting [44].

As the 3’UTR SNPs indeed contribute significantly to the expression variations, researchers utilized the expression association data to support the functionalities of the candidate SNPs that were predicted to alter miRNA bindings. Based on the expression data from Genevar database [52], which contain the significant expression quantitative trait loci (eQTLs) identified in three types of different tissues of twin-pair individuals, Richardson *et al.* shortlisted four candidate SNPs that could contribute to disease phenotypes via altering miRNA target sites and gene expression [12]. Their analysis integrated functional prediction, genotype-expression associations and co-expression of miRNA and targets in the same tissues and provided hypotheses for genetic variants in 3’UTRs affecting disease phenotypes. 

Similar approaches were utilized by Zhang *et al.*, who employed a linear model to assess the effects of polymorphic miRNA target sites on the gene expression phenotypes, which were characterized in the LCLs of HAPMAP individuals. They identified 17 and 9 candidate SNPs that potentially alter miRNA bindings and lead to expression differences in CEU and YRI populations, respectively. As some of the target genes are implicated in diseases or biological traits, these candidate SNPs are likely to contribute to the phenotypic differences [28]. 

The study by Gamazon *et al.* added another layer of complexity by including the inverse correlation between miRNA and their target expression levels in the LCLs of HAPMAP individuals. They first identified a list of negatively correlated miRNA-mRNA pairs, and found the SNPs in the 3’UTRs that showed associations with mRNA expression. Furthermore, they used different prediction algorithms to confirm that the SNPs indeed change the miRA-mRNA interactions. The relationships between SNPs, mRNA expression and miRNA expression strengthened the model, and several SNP-mRNA associations as well as miRNA-mRNA interactions were successfully validated in this study [46]. 

As discussed earlier, in addition to the SNPs in the target sites, miRNA polymorphisms, especially those in the seed regions, could also affect target recognition, and change target gene expression. SNPs within the pre-miRNAs had been tested for associations with mRNA expression by Lu *et al.* [44]. Similar to the 3’UTR SNPs, genotypes of the pre-miRNA polymorphisms were integrated with microarray expression data, 14 genetic variants were identified to be associated with direct or indirect target mRNA expression. It was found that the differences in the target gene expression are mainly associated with SNPs outside seed regions, suggesting that polymorphisms do not directly alter targeting but affecting the abundance of mature miRNAs. Using the miRNA and mRNA expression data in another groups of Caucasian individuals [43], the authors confirmed that differences in target gene expression could be regulated by the expression variations of the miRNAs [44]. 

## 5. Available Resources

Along with the comprehensive genome-wide studies on polymorphisms associated with miRNA regulation, a few databases had been developed to integrate different features of the miRNA-related genetic variants, and facilitate the research in this field. Some early databases, e.g., PolymiRTS [27] mainly included SNPs in the miRNA target sites, while recent updates and new databases also incorporated genetic variants within pre-miRNAs or miRNA-processing genes [13,19]. 

The contents of the miRNA-associated polymorphism databases are summarized into three categories: annotation, functional prediction and expression associations of the miRNA-related genetic polymorphisms. The features of several comprehensive databases are listed in Table 4. Due to the abundance of candidate SNPs in 3’UTRs, nearly all the databases included the information about polymorphic target sites, and utilized different prediction methods to assess their effects on miRNA bindings. On the other hand, SNPs within miRNAs were annotated by miRvar, Patrocles, PolymiRTS 2.0, miRNASNP and MirSNP databases, while the functional effects of these polymorphisms were also incorporated in some of the databases [13,19,24,32,41]. Moreover, Patrocles database annotated the SNPs that affect the coding sequences and splice sites of the miRNA-processing genes, as these variants may alter the gene function and further lead to changes in miRNA processing pathway [19]. 

In addition to SNP annotation and functional prediction, PolymiRTS 2.0 included the eQTL data identified in different human tissues. The database also annotated the SNPs residing in the disease-associated genes as found by genome-wide association study (GWAS), but the associations between SNPs and disease phenotypes had not been validated [24]. As a complementary resource, miRdSNP database incorporated the disease-associated SNPs curated from literature, in addition to the annotation and functional prediction of the 3’UTR SNPs [53]. For SNPs within miRNA loci, disease associations were included in miRvar database [41].

**Table 3:** Summary of Studies on SNPs Associated with MiRNA or Target Gene Expression.

Study	Samples	Expression data	Genotype data	Candidate SNPs
Huang *et al.* [45]	LCLs from 53 CEU and 54 YRI HAPMAP subjects	miRNA microarray data	HapMap release 24	272 SNPs with high population differentiation significantly associated with 33 differentially expressed miRNAs
Gamazon * et al.* [46]				31 genome-wide significant SNPs associated with miRNA expression
	LCLs from 87 CEU and 89 YRI HAPMAP subjects	mRNA microarray data		25 SNPs in CEU and 25 in YRI potentially affect miRNA binding and mRNA expression
Richardson *et al.* [12]	160 fat cell biopsy, 166 LCLs and 160 skin punch biopsy from healthy twins in MuTHER study [49]	mRNA microarray data	Genotype data from MuTHER study	Four SNPs associated with expression and disease phenotypes potentially via altering miRNA target sites
Zhang * et al.* [28]	LCLs from 87 CEU and 89 YRI HAPMAP subjects	mRNA microarray data	HapMap release 27	17 SNPs in CEU and 9 in YRI potentially affect mRNA expression via altering miRNA binding sites
Lu *et al.* [44]	LCLs from 32 CEU, 41 CHB, 39 JPT and 37 YRI HAPMAP subjects	mRNA microarray data	1000 Genomes Project Phase 1	14 genetic variants significantly associated with mRNA expression via direct or indirect targeting

It is notable that there was overlap between different resources, but none of them provided a comprehensive collection of all the polymorphisms that are important for miRNA regulation. Moreover, none of the databases comes along with the updates of the SNP and miRNA databases. Thus, many novel polymorphisms, especially for those identified in the general populations by 1000 genomes project [54], and newly annotated miRNAs in miRBase release 18 and 19, were not incorporated in the majority of the resources. 

## 6. Conclusions

Through a number of genome-wide studies on the polymorphisms involved in miRNA regulation, we have acquired better understanding about how genetic variants can affect miRNA regulation via different ways and contribute to phenotypic changes. Nevertheless, our knowledge in this field is far from complete, and some questions have not been well addressed by the previous studies and require future investigations.

First, different studies applied similar or different approaches to assess whether 3’UTR SNPs alter miRNA target sites, but most of them lacked experimental support. It is unavoidable that many false positives had been be generated, as Chen *et al.* claimed that only about 30-50% SNPs predicted to create novel target sites were functional, even with the evidence of co-expressed miRNA and target genes, which was not considered by some other studies [9]. 

In order to prioritize the candidate polymorphisms from a large number of potentially functional SNPs, expression associations had been used as evidence to support the SNP functionality [12,28,46]. However, it is notable that both miRNA and mRNA eQTLs were mainly identified in the LCLs of CEU and YRI populations, due to the availability of the expression data. It was reported that polymorphisms may affect gene expression levels differently in different cell types [48], thus the current collection of eQTLs were not comprehensive. This may explain why a large number of SNPs that potentially affect miRNA targeting had not been associated with expression phenotypes. Moreover, association studies based on the transcript expression levels did not capture the SNPs that alter miRNA binding sites and lead to change in translational repression and finally protein abundance. Integration of protein expression data in future studies will definitely aid in the studies on the polymorphisms in miRNA regulation.

Second, though Huang *et al.* and Gamzon *et al.* had characterized the polymorphisms associated with miRNA expression, they reported that most of these polymorphisms affected miRNA expression *in trans* [45,46], hence the underlying mechanisms remains a challenge. On the other hand, though Gong *et al.* identified the pre-miRNA SNPs that alter the stability of pre-miRNAs and potentially change the mature miRNA abundance [13], the potential functions of the SNPs at the miRNA loci had not been integrated with the miRNA expression. In this case, the functionality of these polymorphisms had not been fully characterized and validated. 

**Table 4:** Summary of Databases about MiRNA-associated Polymorphisms.

Database	Link	Annotation			Potential functions			Phenotype associations			Reference
		miRNA loci	miRNA targets	miRNA-processing genes	miRNA stability and biogenesis		Gain or loss of target sites	Expression		Diseases	
miRvar	http://genome.igib.res.in/mirlovd	√	×	×	√	N.A.		×	√		[41]
PolymiRTS 2.0	http://compbio.uthsc.edu/miRSNP	√	√	×	×	miRNA seed SNPs, 3'UTR SNPs		√	√		[24]
Patrocles	http://www.patrocles.org/Patrocles.htm	√	√	√	√	3'UTR SNPs		×	×		[19]
miRNASNP	http://www.bioguo.org/miRNASNP	√	√	×	√	miRNA seed SNPs, 3'UTR SNPs		×	×		[13]
dbSMR	http://miracle.igib.res.in/polyreg	×	√	×	×	3'UTR SNPs		×	×		[34]
SNP effects on microRNA targeting	http://www.bigr.medisin.ntnu.no/mirsnpscore	×	√	×	×	3'UTR SNPs		×	×		[35]
miRdSNP	http://mirdsnp.ccr.buffalo.edu/browse-genes.php	×	√	×	×	3'UTR SNPs		×	√		[53]
MirSNP	http://cmbi.bjmu.edu.cn/mirsnp	√	√	×	×	3'UTR SNPs		×	×		[32]

Last but not least, the comprehensive study by Gamazon *et al.* illustrated that target gene expression could be affected by the SNPs in the 3’UTRs, and also correlate with miRNA expression. In addition, the miRNA abundance could also be associated with genetic variants [46]. It suggests that miRNA-mRNA interaction could be affected by multiple genetic polymorphisms. As our current knowledge is still limited to the functions of single SNPs, it would be fascinating to investigate the effects of multiple polymorphisms, and identify their contribution to the phenotypic differences in the near future. 
